# Supplementary figures and images for: Causes of death in children with congenital Zika syndrome in Brazil, 2015 to 2018: A nationwide record linkage study
Source: PLoS Med. 2023 Feb 24;20(2):e1004181. doi: 10.1371/journal.pmed.1004181 (PMC9956022; doi:10.1371/journal.pmed.1004181)

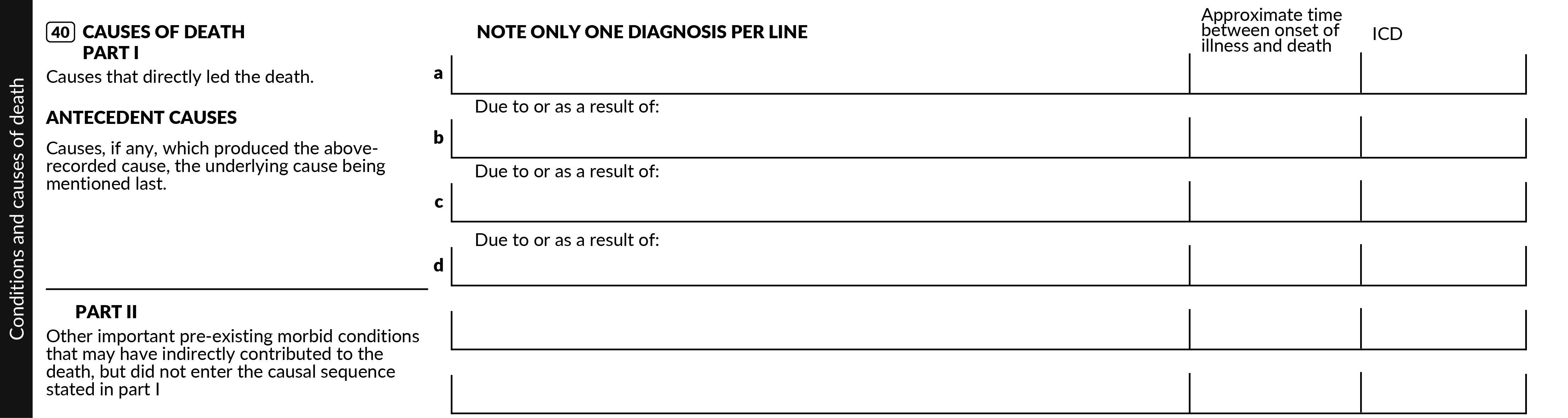

Supplement: S1 Fig — (TIF) [file pmed.1004181.s002.tif]

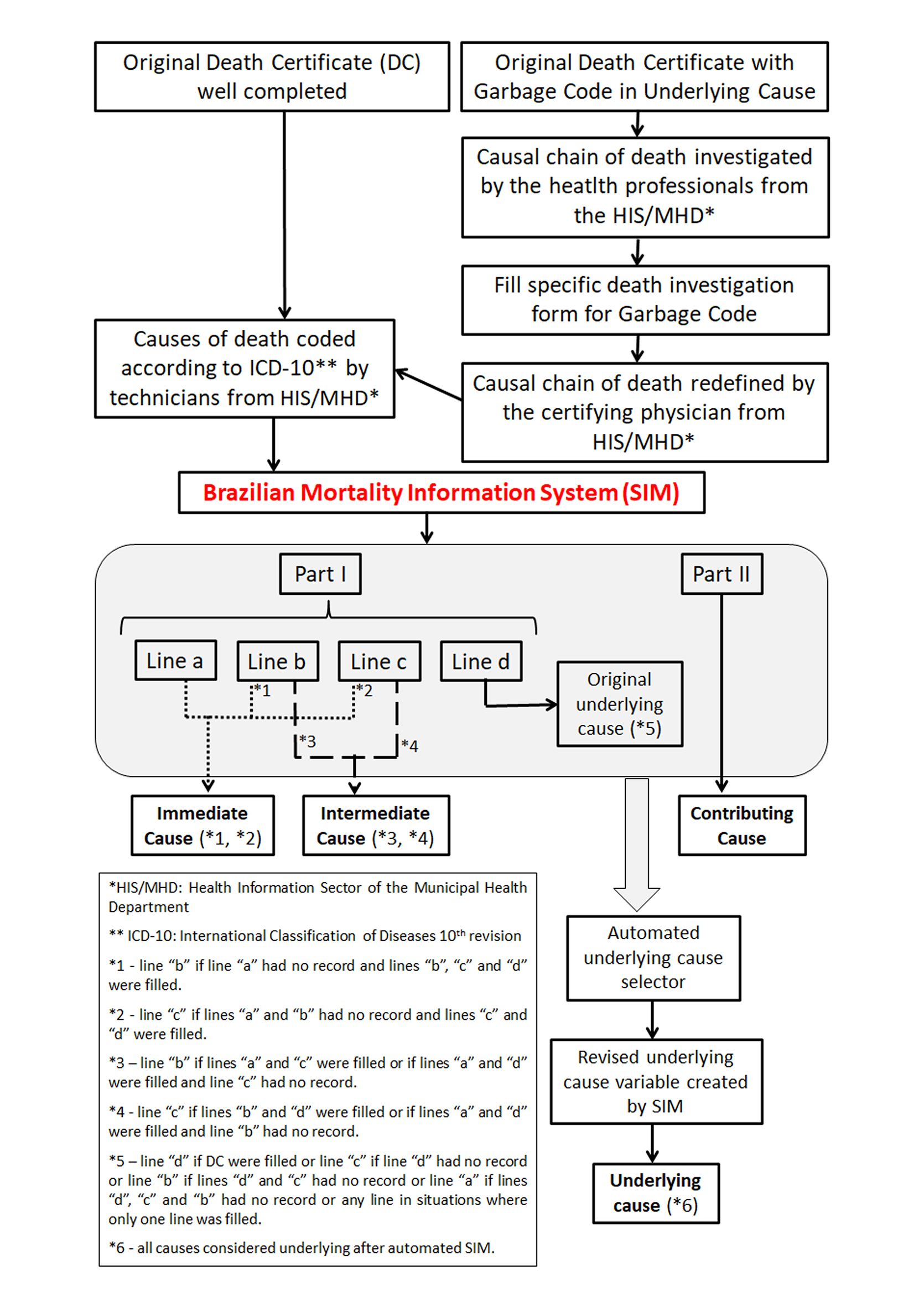

Supplement: S2 Fig — Brazil, 2015 to 2018. (TIF) [file pmed.1004181.s003.tif]

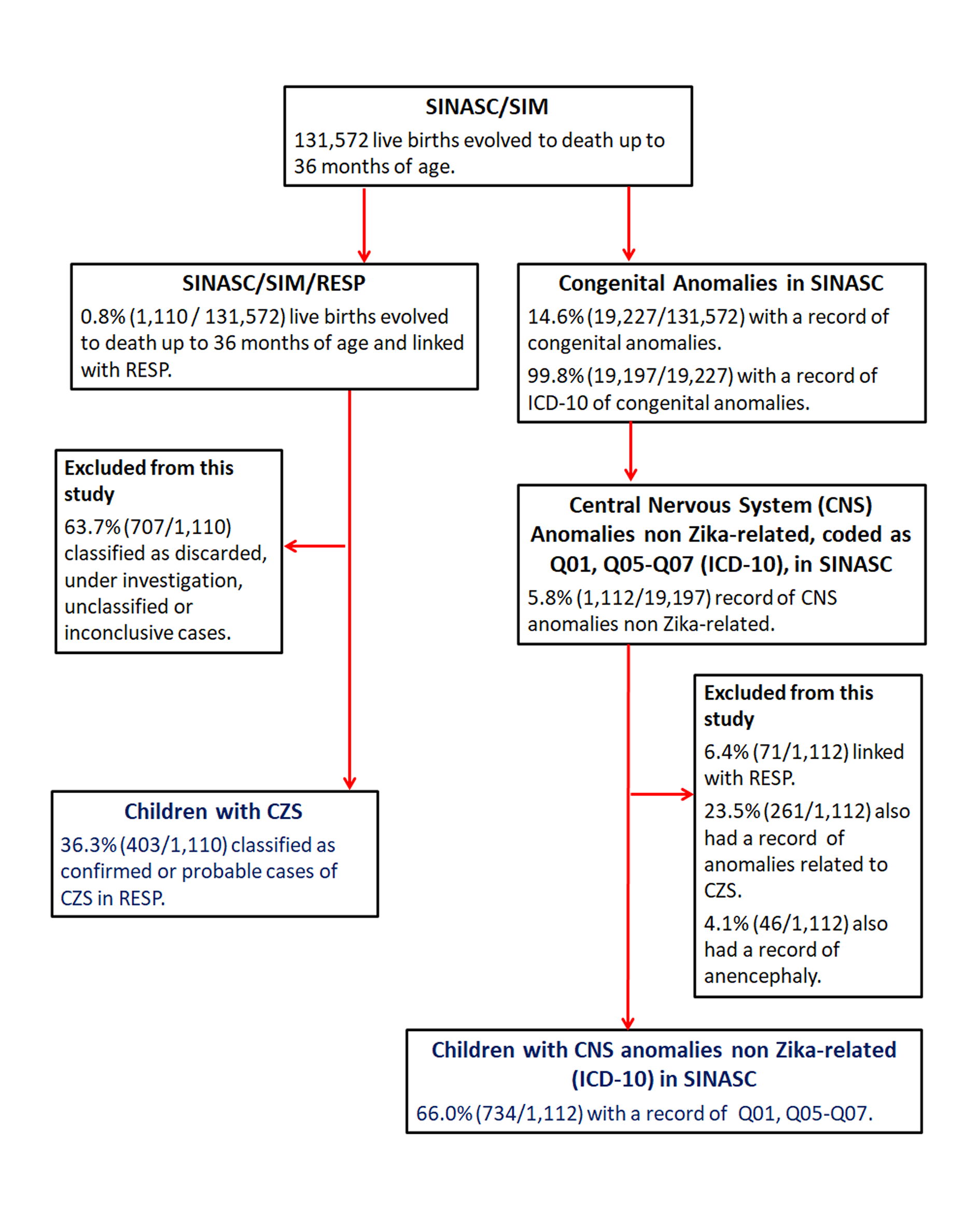

Supplement: S3 Fig — Brazil, 2015 to 2018. (TIF) [file pmed.1004181.s004.tif]
