# Supplementary material for: Causes of death in children with congenital Zika syndrome in Brazil, 2015 to 2018: A nationwide record linkage study
Source: PLoS Med. 2023 Feb 24;20(2):e1004181. doi: 10.1371/journal.pmed.1004181 (PMC9956022; doi:10.1371/journal.pmed.1004181)
Supplement: S1 Table — (DOCX) [file pmed.1004181.s005.docx]

**Table S1.** Main underlying causes of death (Number and Proportional Mortality/PM%) of 403 children up to 36 months of age, born with congenital Zika syndrome (CZS) 2015-2018, values of these indicators for those born with congenital anomalies (CA) of the central nervous system (CNS) non-Zika related, 2012-2013 and proportional mortality ratio between causes (PMRc) according to Groups and Types of causes^1^ in Brazil.

| **Groups and Types of causes^1^** | **CZS**  **(2015-2018)** | | **CA of CNS non-Zika related (2012-2013)** | |  |
| --- | --- | --- | --- | --- | --- |
|  | **N** | **PM(%)** | **N** | **PM(%)** | **PMRc** |
| **Some infectious and parasitic diseases (A00 - B99)** | **35** | **8.7** | **9** | **3.0** | **2.9** |
| A41.9 - Unspecified septicemia | 17 | 4.2 | 6 | 2.0 | 2.1 |
| A92.8 - Other specified viral fevers transmitted by mosquitoes | 6 | 1.5 | - | - | - |
| **Endocrine. nutritional and metabolic diseases (E00-E90)** | **7** | **1.7** | **-** | **-** | **-** |
| 3E43 - Severe protein-calorie malnutrition unspecified | 4 | 1.0 | - | - | - |
| **Nervous System Diseases (G00-G99)** | **20** | **5.0** | **10** | **3.4** | **1.5** |
| G80.9 - Unspecified cerebral palsy | 5 | 1.2 | - | - | - |
| **Respiratory system diseases (J00-J99)** | **18** | **4.5** | **7** | **2.4** | **1.9** |
| J18.9 - Unspecified Pneumonia | 8 | 2.0 | 2 | 0.7 | 2.9 |
| **Some conditions originating in the perinatal period (P00-P96)** | **58** | **14.4** | **32** | **10.8** | **1.3** |
| P00.2 - Fetus and newborn affected by the mother's infectious and parasitic diseases | 7 | 1.7 | 1 | 0.3 | 5.7 |
| P21.9 - Asphyxia at birth unspecified | 4 | 1.0 | - | - | - |
| P22.0 - Newborn respiratory distress syndrome | 3 | 0.7 | 2 | 0.7 | 1.0 |
| P36.9 - Unspecified bacterial septicemia of the newborn | 11 | 2.7 | 9 | 3.0 | 0.9 |
| P37.1 - Congenital toxoplasmosis | 5 | 1.2 | - | - | - |
| **Congenital malformations. deformities and chromosomal anomalies (Q00-Q99)** | **232** | **57.6** | **229** | **77.4** | **0.7** |
| Q00.0 – Anencephaly | 10 | 2.5 | 7 | 2.4 | 1.0 |
| Q01.9 - Encephalocele unspecified | 3 | 0.7 | 40 | 13.5 | 0.1 |
| Q02 – Microcephaly | 93 | 23.1 | - | - | - |
| Q03.1 - Atresia of the Clefts of Luschka and the foramen of Magendie | 4 | 1.0 | 3 | 1.0 | 1.0 |
| Q03.9 - Unspecified congenital hydrocephalus | 9 | 2.2 | 9 | 3.0 | 0.7 |
| Q04.2 – Holoprosencephaly | 4 | 1.0 | - | - | - |
| Q04.3 - Other deformities due to brain reduction | 12 | 3.0 | 3 | 1.0 | 3.0 |
| Q07.9 - Unspecified congenital malformation of the nervous system | 6 | 1.5 | 7 | 2.4 | 0.6 |
| Q24.9 - Unspecified malformation of the heart | 9 | 2.2 | 7 | 2.4 | 0.9 |
| Q33.6 - Lung hypoplasia and dysplasia | 10 | 2.5 | 2 | 0.7 | 3.6 |
| Q89.7 - Multiple congenital malformations not classified elsewhere | 18 | 4.5 | 21 | 7.1 | 0.6 |
| Q89.9 - Multiple congenital malformations not classified elsewhere | 11 | 2.7 | 12 | 4.1 | 0.7 |
| **Symptoms. abnormal signs and findings from clinical and laboratory examinations. unclassified elsewhere (R00-R99)** | **7** | **1.7** | **2** | **0.7** | **2.4** |
| R99 - Other ill-defined and unspecified causes of mortality | 5 | 1.2 | 2 | 0.7 | 1.7 |
| Other groups and types of causes | 26 | 6.5 | 7 | 2.4 | 2.7 |
| **Total** | **403** | **100.0** | **296** | **100.0** | **1.0** |

Source: Center of Data and Knowledge for Health-CIDACS: Linkage of the Live Birth Information System/SINASC. Public Health Events Registry/RESP and Mortality Information System/SIM.^1^ICD 10 (International Classification of Diseases and Causes of Death (ICD 10th Revision).

Only causes of death whose absolute frequency were >3 are included separately.

PM% calculated in relation to the total of causes of death.
