# Supplementary material for: Causes of death in children with congenital Zika syndrome in Brazil, 2015 to 2018: A nationwide record linkage study
Source: PLoS Med. 2023 Feb 24;20(2):e1004181. doi: 10.1371/journal.pmed.1004181 (PMC9956022; doi:10.1371/journal.pmed.1004181)
Supplement: S2 Table — (DOCX) [file pmed.1004181.s006.docx]

**Table S2.** Main intermediate causes of death (Number and Proportional Mortality/PM%) of 403 children up to 36 months of age. born with congenital Zika syndrome (CZS) 2015-2018, values of these indicators for those born with congenital anomalies (CA) of the central nervous system (CNS) non-Zika related, 2012-2013 and proportional mortality ratio between causes (PMRc) according to Groups and Types of causes^1^ in Brazil.

| **Groups and Types of causes^1^** | **CZS**  **(2015-2018)** | | **CA of CNS non-Zika related (2012-2013)** | |  |
| --- | --- | --- | --- | --- | --- |
|  | **N** | **PM(%)** | **N** | **PM(%)** | **PMRc** |
| **Some infectious and parasitic diseases (A00 - B99)** | **35** | **13.5** | **9** | **6.2** | **2.2** |
| A41.9 - Unspecified septicemia | 29 | 11.2 | 6 | 4.1 | 2.7 |
| **Respiratory system diseases (J00-J99)** | **42** | **16.2** | **12** | **8.2** | **2.0** |
| J18.9 - Unspecified pneumonia | 12 | 4.6 | 3 | 2.1 | 2.2 |
| J69.0 - Pneumonitis due to food or vomiting | 5 | 1.9 | 3 | 2.1 | 0.9 |
| J96.0 - Acute breathing insufficiency | 3 | 1.2 | 2 | 1.4 | 0.9 |
| J96.9 - Unspecified respiratory failure | 9 | 3.5 | 1 | 0.7 | 5.0 |
| **Genitourinary system diseases (N00-N99)** | **5** | **1.9** | **4** | **2.7** | **0.7** |
| N17.9 - Acute kidney failure unspecified | 3 | 1.2 | 1 | 0.7 | 1.7 |
| **Some conditions originating in the perinatal period (P00-P96)** | **67** | **25.9** | **55** | **37.7** | **0.7** |
| P07.1 - Other low birth weight newborns | 8 | 3.1 | 3 | 2.1 | 1.5 |
| P07.2 - Extreme immaturity | 3 | 1.2 | - | - | - |
| P21.9 - Asphyxia at birth unspecified | 3 | 1.2 | 1 | 0.7 | 1.7 |
| P22.9 - Unspecified newborn respiratory distress | 4 | 1.5 | - | - | - |
| P23.9 - Unspecified congenital pneumonia | 5 | 1.9 | 2 | 1.4 | 1.4 |
| P28.5 - Newborn respiratory failure | 4 | 1.5 | 6 | 4.1 | 0.4 |
| P36.9 - Unspecified bacterial septicemia of the newborn | 5 | 1.9 | 13 | 8.9 | 0.2 |
| P39.9 - Infection of the perinatal period not specified | 8 | 3.1 | 2 | 1.4 | 2.2 |
| **Congenital malformations. chromosomal deformities and anomalies (Q00-Q99)** | **50** | **19.3** | **23** | **15.8** | **1.2** |
| Q02 – Microcephaly | 23 | 8.9 | 2 | 1.4 | 6.4 |
| Q04.8 - Other specified congenital malformations of the brain | 3 | 1.2 | - | - | - |
| Q07.9 - Unspecified congenital malformation of the nervous system | 4 | 1.5 | - | - | - |
| Q89.9 - Unspecified congenital malformations | 5 | 1.9 | 4 | 2.7 | 0.7 |
| **Abnormal symptoms. signs and findings from clinical and laboratory examinations. unclassified elsewhere (R00-R99)** | **16** | **6.2** | **7** | **4.8** | **1.3** |
| R56.8 - Other and unspecified seizures | 5 | 1.9 | 3 | 2.1 | 0.9 |
| Other groups and types of causes | 44 | 17.0 | 36 | 24.7 | 0.7 |
| **Total** | **259** | **100.0** | **146** | **100.0** | **1.0** |

Source: Center of Data and Knowledge for Health-CIDACS: Linkage of the Live Birth Information System/SINASC. Public Health Events Registry/RESP and Mortality Information System/SIM.^1^ICD 10 (International Classification of Diseases and Causes of Death (ICD 10th Revision).

Only causes of death whose absolute frequency were >3 are included separately.

PM% calculated in relation to the total of causes of death.
