# Supplementary material for: Causes of death in children with congenital Zika syndrome in Brazil, 2015 to 2018: A nationwide record linkage study
Source: PLoS Med. 2023 Feb 24;20(2):e1004181. doi: 10.1371/journal.pmed.1004181 (PMC9956022; doi:10.1371/journal.pmed.1004181)
Supplement: S3 Table — (DOCX) [file pmed.1004181.s007.docx]

**Table S3.** Main immediate causes of death (Number and Proportional Mortality/PM%) of 403 children up to 36 months of age. born with congenital Zika syndrome (CZS) 2015-2018, values of these indicators for those born with congenital anomalies (CA) of the central nervous system (CNS) non-Zika related, 2012-2013 and proportional mortality ratio between causes (PMRc) according to Groups and Types of causes^1^ in Brazil.

| **Groups and Types of causes^1^** | **CZS**  **(2015-2018)** | | **CA of CNS non-Zika related (2012-2013)** | |  |
| --- | --- | --- | --- | --- | --- |
|  | **N** | **PM(%)** | **N** | **PM(%)** | **PMRc** |
| **Some infectious and parasitic diseases (A00 - B99)** | **41** | **12.4** | **24** | **11.4** | **1.1** |
| A41.9 - Unspecified septicemia | 39 | 11.8 | 22 | 10.5 | 1.1 |
| **Circulatory System Diseases (I00-I99)** | **7** | **2.1** | **6** | **2.9** | **0.7** |
| I50.9 - Unspecified heart failure | 4 | 1.2 | - | - | - |
| **Respiratory System Diseases (J00-J99)** | **63** | **19.1** | **25** | **11.9** | **1.6** |
| J18.0 - Unspecified bronchopneumonia | 5 | 1.5 | 1 | 0.5 | 3.0 |
| J18.9 - Unspecified bronchopneumonia | 6 | 1.8 | 2 | 1.0 | 1.8 |
| J80 - Adult Respiratory Distress Syndrome | 5 | 1.5 | - | - | - |
| J96.0 - Acute breathing insufficiency | 24 | 7.3 | 9 | 4.3 | 1.7 |
| J96.9 - Unspecified respiratory failure | 16 | 4.8 | 8 | 3.8 | 1.3 |
| **Some conditions originating in the perinatal period (P00-P96)** | **110** | **33.3** | **103** | **49.0** | **0.7** |
| P07.3 - Other preterm newborns | 3 | 0.9 | 1 | 0.5 | 1.8 |
| P21.9 - Asphyxia at birth unspecified | 7 | 2.1 | 4 | 1.9 | 1.1 |
| P26.9 - Unspecified pulmonary hemorrhage originating in the perinatal period | 5 | 1.5 | - | - | - |
| P28.5 - Newborn respiratory failure | 40 | 12.1 | 31 | 14.8 | 0.8 |
| P29.1 - Neonatal cardiac dysrhythmia | 4 | 1.2 | 8 | 3.8 | 0.3 |
| P36.9 - Unspecified bacterial septicemia of the newborn | 14 | 4.2 | 16 | 7.6 | 0.6 |
| P60 - Disseminated intravascular coagulation of the fetus and newborn | 3 | 0.9 | 2 | 1.0 | 0.9 |
| P96.8 - Other specified conditions originating in the perinatal period | 6 | 1.8 | 8 | 3.8 | 0.5 |
| **Congenital malformations. chromosomal deformities and anomalies (Q00-Q99)** | **20** | **6.1** | **10** | **4.8** | **1.3** |
| Q02 - Microcephaly | 12 | 3.6 | - | - | - |
| Q89.7 - Multiple congenital malformations not classified elsewhere | 3 | 0.9 | 1 | 0.5 | 1.8 |
| **Abnormal symptoms. signs and findings from clinical and laboratory examinations. unclassified elsewhere (R00-R99)** | **61** | **18.5** | **16** | **7.6** | **2.4** |
| R09.2 - Respiratory failure | 8 | 2.4 | 3 | 1.4 | 1.7 |
| R57.0 - Cardiogenic shock | 5 | 1.5 | 2 | 1.0 | 1.5 |
| R57.8 - Other forms of shock | 9 | 2.7 | 1 | 0.5 | 5.4 |
| R57.9 - Unspecified shock | 5 | 1.5 | - | - | - |
| R68.8 - Other specified general symptoms and signs | 15 | 4.5 | 5 | 2.4 | 1.9 |
| R99 - Other ill-defined and unespecified causes of mortality | 8 | 2.4 | 2 | 1.0 | 2.4 |
| **Injuries. poisonings and some other consequences of external causes (S00-T98)** | **10** | **3.0** | **11** | **5.2** | **0.6** |
| T17.9 - Foreign body in the respiratory tract unspecified part | 4 | 1.2 | 3 | 1.4 | 0.9 |
| Other groups and types of causes | 18 | 5.5 | 15 | 7.1 | 0.8 |
| **Total** | **330** | **100.0** | **210** | **100.0** | **1.0** |

Source: Center of Data and Knowledge for Health-CIDACS: Linkage of the Live Birth Information System/SINASC. Public Health Events Registry/RESP and Mortality Information System/SIM.^1^ICD 10 (International Classification of Diseases and Causes of Death (ICD 10th Revision).

Only causes of death whose absolute frequency were >3 are included separately.

PM% calculated in relation to the total of causes of death.
