# Supplementary material for: Causes of death in children with congenital Zika syndrome in Brazil, 2015 to 2018: A nationwide record linkage study
Source: PLoS Med. 2023 Feb 24;20(2):e1004181. doi: 10.1371/journal.pmed.1004181 (PMC9956022; doi:10.1371/journal.pmed.1004181)
Supplement: S4 Table — (DOCX) [file pmed.1004181.s008.docx]

**Table S4.** Main contributing causes of death (Number and Proportional Mortality/PM%) of 403 children up to 36 months of age. born with congenital Zika syndrome (CZS) 2015-2018, values of these indicators for those born with congenital anomalies (CA) of the central nervous system (CNS) non-Zika related, 2012-2013 and proportional mortality ratio between causes (PMRc) according to Groups and Types of causes^1^ in Brazil.

| **Groups and Types of causes^1^** | **CZS**  **(2015-2018)** | | **CA of CNS non-Zika related (2012-2013)** | |  |
| --- | --- | --- | --- | --- | --- |
|  | **N** | **PM(%)** | **N** | **PM(%)** | **PMRc** |
| **Some infectious and parasitic diseases (A00 - B99)** | **21** | **6.0** | **-** | **-** | **-** |
| A41.9 - Unspecified septicemia | 3 | 0.9 | - | - | - |
| A92.8 - Other specified viral fevers transmitted by mosquitoes | 11 | 3.2 | - | - | - |
| **Doenças endócrinas. nutricionais e metabólicas (E00-E90)** | **14** | **4.0** | **-** | **-** | **-** |
| E43 - Severe protein-calorie malnutrition unspecified | 3 | 0.9 | - | - | - |
| E46 - Unspecified protein-calorie malnutrition | 6 | 1.7 | - | - | - |
| **Nervous System Diseases (G00-G99)** | **27** | **7.8** | **3** | **2** | **3.9** |
| G40.4 - Other epilepsies and generalized epileptic syndromes | 3 | 0.9 | - | - | - |
| G40.9 - Epilepsy. unspecified | 3 | 0.9 | - | - | - |
| G80.9 - Unspecified cerebral palsy | 3 | 0.9 | - | - | - |
| G91.9 -Unspecified hydrocephalus | 8 | 2.3 | 2 | 1.3 | 1.8 |
| G93.4 - Unspecified encephalopathy | 3 | 0.9 | - | - | - |
| **Genitourinary system diseases (N00-N99)** | **4** | **1.1** | **-** | **-** | **-** |
| N17.9 - Acute kidney failure unspecified | 3 | 0.9 | - | - | - |
| **Some conditions originating in the perinatal period (P00-P96)** | **77** | **22.1** | **58** | **37.9** | **0.6** |
| P00.2 - Fetus and newborn affected by the mother's infectious and parasitic diseases | 10 | 2.9 | 3 | 2.0 | 1.5 |
| P07.0 - Very low birth weight newborn | 3 | 0.9 | 4 | 2.6 | 0.3 |
| P07.1 - Other low birth weight newborns | 12 | 3.4 | 10 | 6.5 | 0.5 |
| P07.3 - Other preterm newborns | 7 | 2.0 | 9 | 5.9 | 0.3 |
| P35.8 - Other congenital viral diseases | 4 | 1.1 | - | - | - |
| P36.9 - Unspecified bacterial septicemia of the newborn | 3 | 0.9 | 2 | 1.3 | 0.7 |
| P37.1 - Congenital Toxoplasmosis | 4 | 1.1 | - | - | - |
| P90 - Newborn seizures | 3 | 0.9 | - | - | - |
| **Congenital malformations. chromosomal deformities and anomalies (Q00-Q99)** | **174** | **50.0** | **82** | **53.6** | **0.9** |
| Q02 - Microcephaly | 76 | 21.8 | 2 | 1.3 | 16.8 |
| Q03.1 - Atresia of the Clefts of Luschka and the foramen of Magendie | 4 | 1.1 | 3 | 2.0 | 0.6 |
| Q03.9 - Unspecified congenital hydrocephalus | 6 | 1.7 | 9 | 5.9 | 0.3 |
| Q04.9 - Unspecified congenital malformation of the brain | 3 | 0.9 | 1 | 0.7 | 1.3 |
| Q21.1 - Atrial septal defect | 3 | 0.9 | 1 | 0.7 | 1.3 |
| Q24.9 - Unspecified malformation of the heart | 6 | 1.7 | 4 | 2.6 | 0.7 |
| Q74.3 - Multiple congenital arthrogryposis | 3 | 0.9 | - | - | - |
| Q89.7 - Multiple congenital malformations. not classified elsewhere | 12 | 3.4 | 4 | 2.6 | 1.3 |
| Q89.9 - Unspecified congenital malformations | 11 | 3.2 | 2 | 1.3 | 2.5 |
| Q99.9 - Unspecified chromosomal abnormality | 3 | 0.9 | 1 | 0.7 | 1.3 |
| Other groups and types of causes | 31 | 8.9 | 10 | 6.5 | 1.4 |
| **Total** | **348** | **100.0** | **153** | **100.0** | **1.0** |

Source: Center of Data and Knowledge for Health-CIDACS: Linkage of the Live Birth Information System/SINASC. Public Health Events Registry/RESP and Mortality Information System/SIM.^1^ICD 10 (International Classification of Diseases and Causes of Death (ICD 10th Revision).

Only causes of death whose absolute frequency were >3 are included separately.

PM% calculated in relation to the total of causes of death.
