# Supplementary material for: Causes of death in children with congenital Zika syndrome in Brazil, 2015 to 2018: A nationwide record linkage study
Source: PLoS Med. 2023 Feb 24;20(2):e1004181. doi: 10.1371/journal.pmed.1004181 (PMC9956022; doi:10.1371/journal.pmed.1004181)
Supplement: Attachment — Submitted filename: point by point EP.docx [file pmed.1004181.s009.docx]

| **Reviewers** | **Answers** |
| --- | --- |
| Please rearrange your title such that it reads: ‘Causes of Death in Children with Congenital Zika Syndrome in Brazil, 2015-2018.: A nationwide **Record-Linkage** study.’ | Done. |
| Please structure your abstract using the PLOS Medicine headings (Background, Methods and Findings, Conclusions). | Done. |
| Abstract Methods and Findings: * Please ensure that all numbers presented in the abstract are present and identical to numbers presented in the main manuscript text. | Done. |
| * In the last sentence of the Abstract Methods and Findings section, please describe the main limitation(s) of the study's methodology. | Done. We included this information in the Abstract Methods. The main limitations of this study were the use of a secondary database without additional clinical information and potential misclassification. |
| Include a short, non-technical Author Summary of your research to make findings accessible to a wide audience that includes both scientists and non-scientists. | Done. We included the Author Summary after the abstract, lines 102-145. |
| Please add the following statement, or similar, to the Methods: "This study is reported as per the REporting of studies Conducted using Observational Routinely-collected health Data (RECORD) Statement (S1 Checklist)." | Done. We included this information in the Methods, lines 177-179. |
| Did your study have a prospective protocol or analysis plan? Please state this (either way) early in the Methods section. | A written analysis plan was not developed; however, all analyses were presented and discussed during regular study group meetings. We included this information in the Methods. |
| The ‘Contributors’, ‘Declarations’ and ‘Funding’ sections can all be removed from the end of the main text – this information is captured via the Submission Form. | Done. |
| References: Journal name abbreviations should be those found in the National Center for Biotechnology Information (NCBI) databases. | Done. |
| **Reviewer 1** | **Answers** |
| Was any information about the children (particularly age distribution) in Zika vs. non-Zika available? This would be helpful to include in the manuscript. | Thank you very much for this suggestion. We included information about the age distribution in the non-Zika group in the manuscript results section. |
| I would add confidence intervals for PMRC (proportional mortality ratio by cause) where a cause of death category for both CZS and the non-Zika has at least 5 deaths in each. Similarly for the calculation of rate of cause of death as underlying cause vs. anywhere in causal sequence. I would also consider reformulating the data used to calculate mortality odds ratio instead of PMRC (Am J Epidemiol 1981; 114: 144-148.), it can avoid bias when there are influences on the underlying denominators. | We appreciated your comment about the confidence intervals for PMRc. However, we did not added the confidence intervals because our study included the entire population, that is, it was not based on samples (<https://www.ncbi.nlm.nih.gov/pmc/articles/PMC5811238/>).  We also appreciated your comment about the analysis in table 5. However, in this table we did not calculate the PMRc. We assessed the “attribution gain” (shown as x-fold increases) for each cause of death when a causal chain assessment was used for each death by aggregating deaths and comparing with the number of deaths that would be attributed to each cause when relying only on underlying condition.  We reformulated the methods and results to make this clear. |
| P5, L135. Are these type of congenital abnormalities (or congenital abnormalities) a notifiable disease in Brazil (i.e. legally required to report any incidence)? I had a look at the sites in the references but google translate did not seem to do a good job translating Portuguese to English. | In Brazil there is a compulsory registration of all congenital abnormalities identified at birth in the live births information system (SINASC).  In addition, due to its Public Health relevance, congenital Zika syndrome cases must also be registered in a specific online form called Public Health Event Records (RESP). |
| P12, L269. I would consider dropping the 'chapter' column here | Thank you. We excluded the chapter column from all tables. |
| **Reviewer 2** | **Answers** |
| How can one be sure that the CNS abnormalities of the control group were definitely not due to Zika if these children born during the Zika epidemic period in Brazil?  A comparator group of children born during the pandemic should not be used given the difficulties in establishing a definitive diagnosis. A pre Zika epidemic comparator group would be more suitable. | Thank you for your comment. We understand your point and performed a sensitivity analysis using as a comparison group children born before the Zika epidemic in Brazil, in 2012-2013, with record of CNS anomalies non-Zika-related (Q01, Q05-Q07 - ICD-10). The results were similar to the main analyses for most of the causes of death. Therefore, we presented these results as supplementary material. We included this information in the Methods, results section, and discussion section.  We decided to exclude Q00 for all comparison group (2015-2018 and 2012-2013) because children with anencephaly die soon after birth. |
| It would be more interesting for the authors to focus on children in the Zika cohort only and show what factors distinguished children with CZS who died by 36 months of age with children with CZS who did not die by this age group. This would be more helpful clinically because it would highlight areas that medical providers need to focus on. They could continue to describe the cascade of diagnoses that led to  death but without trying to compare it to the other group, but instead, showing what features were risk factors for death by 36 months in comparison to children with CZS who did not die. The data is available in their database. The other option is to use a comparator group of the same age but recruited before the Zika epidemic, however an analysis of risk factors leading to death in CZS children only and then the inclusion of the sequence of events that led to death in CZS children would be more informative to readers. | Our research team has already published other articles on CZS addressing the case fatality rate and child-maternal characteristics of cases and deaths related to CZS, as well as its associated factors, in Brazil, from 2015 to 2017 (<https://pubmed.ncbi.nlm.nih.gov/33138282/>) and also on mortality risk and causes of deaths among live births with CZS compared with those without CZS (<http://www.ncbi.nlm.nih.gov/pubmed/35196428>).  However, when developing this study, in addition to describing the causal sequence, our interest was also to verify whether or not these children were more vulnerable to death from some specific causes. |
| Another important consideration is that there are some children with CZS who appear to have other diagnoses which could lead to the CNS findings reported. For example there are 5 children with congenital toxoplasmosis reported in Table 1 in the CZS group. If children have a diagnosis of congenital toxoplasmosis, another congenital infection, they should be excluded from the CZS group because their neurologic manifestations can be attributed to another cause. In the same way, any child with a diagnosed genetic defect or chromosomal abnormality should not be part of the CZS group, these should be excluded. | Thank you. In the CZS group, we maintained the final classification criteria provided by the Brazilian Ministry of Health. This may have led to misclassification of CZS. We included this as a potential limitation of this study in the discussion. |
| Specific comments: Should proofread the manuscript for English corrections. | Thank you very much. Native English speakers have proofread the paper. |
| The abstract should mention causes of death were abstracted from death certificates using the WHO death certificate template. | Thank you. We included this information in the abstract. |
| Abstract should make it clear that the inclusion criteria for both CZS and non-CZS children was death by 36 months of age, because it is not clear. | Thank you. We included this information in the abstract. |
| Conclusion of the abstract: not clearly supported by the results provided since data for the comparator group is not provided in the abstract and there are no statistical analyses included to demonstrate that they were significantly different. | Thank you. We included this information in the abstract. |
| Line 252: 2434 of how many? Per Figure 3 the denominator would be 19197, or 12.7%. | Of the 1,112 children who had records of anomalies of the central nervous system not related to zika, coded as Q01, Q05-Q07 (ICD-10) at SINASC, we excluded from the study who also had records of anencephaly, or ICD-10 related to CZS or children that were linked with RESP. Thus, 734/1,112 were included in the comparison group. We included this information in the Figure 3, in the case definition/methods section, and in the results section. |
| Line 407: Children with CZS may be born small for gestational age, which contributes to the malnutrition. They may have seizures which can also lead to aspiration pneumonia, in addition to dysphagia. | Thank you. We included this information in the discussion. |
| **Reviewer 3** | **Answers** |
| It is an unambitious study and based on secondary data from health information systems, therefore, subject to problems related to the criteria for defining cause of death and quality of filling out death certificates. Even so, I think it is important to be published as a way of documenting the findings related to the country most affected by this disease. Unless I am mistaken, this is the largest number of deaths associated with the Zika Virus ever published and therefore a document of historical value and will allow monitoring of CZS cases and their complications. | Thank you. |
