# Supplementary material for: Causes of death in children with congenital Zika syndrome in Brazil, 2015 to 2018: A nationwide record linkage study
Source: PLoS Med. 2023 Feb 24;20(2):e1004181. doi: 10.1371/journal.pmed.1004181 (PMC9956022; doi:10.1371/journal.pmed.1004181)
Supplement: Attachment — Submitted filename: Point by point (4).docx [file pmed.1004181.s010.docx]

**Causes of Death in Children with Congenital Zika Syndrome in Brazil, 2015-2018.: A nationwide Record Linkage Study.**

| **Editor** | **Answers** |
| --- | --- |
| Abstract Conclusions: Please avoid vague statements such as "reflects important aspects of CZS”. Mention only specific implications substantiated by the results | Done. We excluded this information in the Abstract Conclusions. |
| Please trim the 7th bullet point in your Author Summary – ideally, bullet points should only run to 1-2 sentences. | Done.We trim the 7th bullet point in our Author Summary. |
| Line 153: Please combine these citations (i.e., [2, 3]). | Done. |
| Line 179: I could not locate the RECORD checklist in the Supporting Information, please include the completed checklist (when completing the checklist, please use section headings and paragraph numbers, rather than line/page numbers). | Done. We included the complete checklist in the Supporting Information. |
| Please ensure you have defined all abbreviations in Table and Figure legends (including in the Supporting Information). | Done. |
| Lines 266-271: This sentence is both long and quite confusing – please check for clarity. | Done. We split the sentences into two paragraphs and revised the text, lines 270-284. |
| Line 284: Sensitivity analysis. | Done. |
| Please confirm that no informed consent was required for this study. | Done. We included this information in Ethics Statement section, line 301. |
| Please ensure that any changes in the analysis- including those made in response to peer review comments-- are identified as such in the Methods section of the paper, with rationale. | Done. |
| The legends of Tables 1-4 and S1-S4 contain a flag (1) but I could not locate the corresponding flag in the tables. | Done. We added a flag ^1^ in all Tables. |
| Lines 452-454: This sentence does not scan properly, please confirm there are no omissions. | Done. |
| Line 496: Please temper claims to primacy by including ‘to our knowledge’, or similar. | Done. |
| Reference 4 contains a flag (†), please delete. | Done. |
| Line 420: Please report Results in the past tense (‘we observed’). | Done. |
